# Cross-cultural adaptation and determination of the reliability and validity of PRTEE-S (Patientskattad Utvärdering av Tennisarmbåge), a questionnaire for patients with lateral epicondylalgia, in a Swedish population

**DOI:** 10.1186/1471-2474-9-79

**Published:** 2008-06-05

**Authors:** Pia Nilsson, Amir Baigi, Bertil Marklund, Jörgen Månsson

**Affiliations:** 1 Tvååker Primary Health Care Centre, Varberg, Sweden; 2 Research and Development Unit, Primary Health Care Halland, Falkenberg, Sweden; 3 Department of Public Health and Community Medicine/Primary Health Care, University of Gothenburg, Sweden

## Abstract

**Background:**

In Sweden, as well as in Scandinavia, there is no easy way to evaluate patients' difficulties when they suffer from lateral epicondylitis/epicondylalgia. However, there is a Canadian questionnaire, in English, that could make the evaluation of a patient's pain and functional loss both quick and inexpensive. Therefore, the aim of this study was to translate and cross-culturally adapt the questionnaire "Patient-rated Tennis Elbow Evaluation" into Swedish (PRTEE-S; "Patientskattad Utvärdering av Tennisarmbåge"), and to evaluate the reliability and validity of the test.

**Methods:**

The Patient-rated Tennis Elbow Evaluation was cross-culturally adapted for the Swedish language according to well-established guidelines. Fifty-four patients with unilateral epicondylitis/epicondylalgia were assessed using the PRTEE-S (Patientskattad Utvärdering av Tennisarmbåge), the Disabilities of Arm, Shoulder, and Hand questionnaire, and the Roles & Maudsley score to establish the validity and reliability of the PRTEE-S. Reliability was determined via calculation of the intra-class correlation coefficient (ICC) the internal consistency was assessed by Cronbach's alpha, and validity was calculated using Spearman's correlation coefficient.

**Results:**

The test-retest reliability, using the PRTEE-S (Patientskattad Utvärdering av Tennisarmbåge) intraclass correlation coefficient, was 0.95 and the internal consistency was 0.94. The PRTEE-S correlated well with the Disabilities of the Arm, Shoulder, and Hand questionnaire (r = 0.88) and the Roles & Maudsley score (r = 0.78).

**Conclusion:**

The PRTEE-S (Patientskattad Utvärdering av Tennisarmbåge) represents a reliable and valid instrument to evaluate the subjective outcome in Swedish speaking patients with lateral epicondylitis/epicondylalgia, and can be used in both research and clinical settings.

## Background

Lateral epicondylitis/epicondylalgia is a common diagnosis in most populations, with a prevalence of 2% [1]. Older literature refers to this diagnosis as lateral epicondylitis, but as there are studies that support the fact that there is no ongoing inflammation in the tendons, it may be more correct to call this disease epicondylalgia [2,3]. However, what the disease really means to patients is pain in the area of the lateral epicondyle, resulting in loss of function. Although this disease is very common, is no definitive way to cure it. Many authors have described various treatments [4-7], but there has never been a real consensus regarding what to do with this group of patients. It has been shown that there is a connection between the work environment and lateral epicondylalgia [8]. Men and women are equally affected, although there is a difference in how they are affected. Men are more affected when they perform monotonous work with a small grip; women are affected when they perform monotonous work, and even more strongly affected if they have poor social support at work [9].

If there were a tool for early detection of a patient's pain and functional loss, it could be a fast way to determine the best course of treatment. The tool also has to be sensitive, so that it can easily detect whether the treatment is effective. The questions should also be designed for rapid completion and easy understanding. In Canada in 1999, the first questionnaire was developed (which only considered the lateral elbow). This questionnaire was called the Patient-rated Forearm Evaluation Questionnaire (PRFEQ) [10] and was generated in a similar fashion the scale for Patient-rating of Wrist and Disability[11]. JC MacDermid was the developer of the PRFEQ, which was first published and used for a master's thesis by Jen Wuori (supervisor JC MacDermid; the main thesis on bracing for tennis elbow was published later [12]. Dr Tom Overend, a committee member, was the first to publish the reliability of the scale [10]. To assist with tool construction, the authors did a literature review, in which they looked at the physical requirements for performing a variety of functional activities and studies that had used standard patient questionnaires to evaluate the two basic outcomes: pain and function. The PRFEQ was based on two sources: Stratford et al. [13] and the wrist questionnaire mentioned above [11], which was used at the Hand and Upper Limb Centre at St. Joseph's Health Centre in London. The questionnaire assessed the patient's subjective pain and functional disability for the previous week. It took only five minutes to complete the questionnaire, which provided a very quick way to assess the patients' experiences regarding their elbow disease [10]. In 2005, the PRFEQ was considered to be the most reliable, reproducible and change-sensitive questionnaire that concerned the lateral epicondyle. In this study, the PRFEQ was compared to the Visual Analogue Scale (VAS), the Disabilities of the Arm, Shoulder, and Hand questionnaire, the Medical Outcomes Study 36-item Short Form Health Survey and the pain-free grip strength measurement. Newcomer et al. recommended that the PRFEQ should be used as a standard outcome measure in research on lateral epicondylalgia [14]. In 2005, the PRFEQ was updated slightly by the developer JC MacDermid to accommodate findings from different research groups and to improve clarity. Some words were changed so that it could be used all over the world. For example, the question concerning the function "carrying a grocery bag" was updated to "carrying a grocery bag or a brief-case by the handle" which is a more up-to-date question and may even apply better to both genders [15]. The scoring of this questionnaire is consistent with the Patient-Wrist Evaluation and Patient-Elbow Evaluation. This questionnaire, in order to not be misleading in the desired outcome, was now called the Patient-Tennis Elbow Evaluation. In 2007, this updated version was validated and considered to be reliable for this disease [16].

There is always difficulty in comparing studies when different measures are used. A universally used clinical outcome, based on this questionnaire, would make it easier to compare the effects of treatment and possibly facilitate the decision making regarding the best way to treat patients. The PRFEQ was translated into Hong Kong Chinese [17], and as the updated PRTEE version has already been written in English, translating it into Swedish would make for a third language and would serve as a way to spread this form of evaluation throughout Scandinavia. To the authors' knowledge, there is no such questionnaire in Sweden. Therefore, the purpose of this study was to perform a translation and cross-cultural adaptation of the updated version of the PRFEQ, i.e., the PRTEE questionnaire, into Swedish, in order to analyze the structure of the questionnaire and to perform reliability and validity evaluations of the Swedish version (PRTEE-S; Patientskattad Utvärdering av Tennisarmbåge) (Additional file 1).

## Methods

### Cross-cultural adaptation

#### Questionnaire

The original PRFEQ (Patient-rated Forearm Evaluation Questionnaire) was developed from two sources and assessed for reliability, validity, and sensitivity of the visual analogue scales of pain and function. The instrument was highly reliable, moderately valid and very sensitive to changes [13]. In 2007, the PRFEQ was updated and became the PRTEE (Patient-rated Tennis Elbow Evaluation) [15,16]. The PRTEE estimates the patient's pain and function over the past week. The questionnaire consists of 15 questions: the first five questions concern the pain in the elbow/elbows and the remaining ten questions concern the function of the elbow or elbows. Both the pain and function scales have 11 degrees, starting at 0 and ending at 10, in which 0 is no pain or difficulty in performing a task, and 10 means the worst pain imaginable or the complete inability to perform a task. The total score is the combined score for all questions, including both pain and function. The range is from 0 (no pain or difficulty in performing the task) to 50 (worst pain imaginable) in the pain section, and 100 (inability to use the elbow or elbows) for the function section; the value for the function section is then divided by 2 so that the maximum score is 50. If the task is never performed, the patient is asked in writing to draw a line in the question area.

#### Translation and cross-cultural adaptation

Permission was granted by the developer Dr JC MacDermid.

The translation and cross-cultural adaptation took place in five stages as recommended by Beaton et al [18].

##### The first stage

The first stage is adaptation in the forward translation. Three translators translated the PRTEE from English to Swedish.

##### The second stage

Syntheses of the translations were performed by three other individuals. This was accompanied by a written report documenting the synthesis process, any uncertainties, and how these uncertainties were resolved. All of the translators' solutions were taken into consideration when performing the syntheses.

##### The third stage

A back translation was made from Swedish into English. Working from the synthesized version of the questionnaire, and totally blind to the original version of the PRTEE, three persons translated the questionnaire back into the English language.

##### The fourth stage

A consensus of the back translations was performed by an expert committee of five persons. All of the previous translators' versions of the PRTEE were taken into consideration. The committee reviewed every detail and every discrepancy among the previous translators and performed a pre-final version of the PRTEE-S. Beaton suggested four equivalences to be checked: Semantic equivalence, that the words should have only one meaning so as not to confuse the patients; grammar; idiomatic equivalence, or a check of all the colloquialisms, which turned out to not be an issue; and experiential equivalence, meaning that the items and experiences of daily life were checked and that the language was adapted. For example, a question concerning "turning a doorknob" does not work in Sweden, where there are no such doorknobs. The correct task would be "turning a door handle or a key". Finally, conceptual equivalence was verified by checking the original PRTEE and the back-translated questionnaires for all equivalences. The translators in the expert committee had to make sure that the final questionnaire would be understood by the equivalent of a 12-year-old (Grade 6 reading level) as is the general recommendation for questionnaires [19].

##### The fifth stage

The final stage of this cross-cultural adaptation and translation process was the pre-test of the pre-final PRTEE, also referred to as face validity. Ten healthy persons and ten persons with the diagnosis of lateral epicondylitis were tested with the pre-final PRTEE, which was now called the PRTEE-S (Patientskattad Utvärdering av Tennisarmbåge). Each of the volunteers completed the questionnaire and was asked if there were any words or sentences that were difficult to understand. For each question, they were asked what they thought the question meant. Both the meaning of the items and the tasks and the chosen response were discussed. This ensured that the pre-final version still retained adequate equivalence in purpose. All of the questions were considered to be easy to understand by all the participants who filled out the questionnaire. There were no words that were difficult to understand, nor any sentences that did not seem adequate to fit the types of symptoms or functional problems of lateral epicondylitis/epicondylalgia.

### Reliability and validity

#### Study group

Physiotherapists, occupational therapists, and general practitioners at eight different health care centres in the south-west of Sweden asked patients with unilateral epicondylitis/epicondylalgia if they were willing to participate in this study. All of the patients provided oral and written informed consent for this study.

### Questionnaires

The PRTEE-S (Patientskattad Utvärdering av Tennisarmbåge) and the DASH (Disabilities of the Arm, Shoulder and Hand) questionnaire [20-22] were filled out twice within 30 minutes. The Roles & Maudsley physical score [23] was provided by the physiotherapist, occupational therapist, or general practitioner who had met with the patient. The DASH is a validated questionnaire designed to measure upper limb disabilities and symptoms [22]. It uses a single-scale, 30-item questionnaire of upper extremity function and symptoms. The minimum sum score is 30 points; the maximum score is 150 points. The DASH score is calculated as the total score minus 30 and then divided by 1.2. The Roles & Maudsley score has four gradations: excellent, meaning no pain, full movement, full activity; good, meaning occasional discomfort, full movement, full activity; fair, meaning some discomfort after prolonged activity; and poor, meaning pain and limited activities [23].

### Statistical analysis

The test-retest reliability for the PRTEE-S (pain, function, overall score) was determined by calculation of the intra-class correlation coefficient (ICC). Variance components for calculation of the ICC were interpreted on the basis of the subjective categories described by Fleiss [24]. ICC s of 0.00 to 0.40 was considered to be "poor", 0.40 to 0.75 "fair to good", and greater than 0.75 "excellent". The internal consistency of the questionnaire was determined by Cronbach's alpha. The criterion/construct (convergent) validity of the PRTEE-S was determined by analysing the relationship between PRTEE-S scores and the scores from the DASH questionnaire and the Roles & Maudsley test using Spearman's correlation coefficient. All of the statistical analyses were carried out using SPSS 15.0 for Windows. Statistical significance was accepted at the 5% level.

### Ethics

This study was approved by the Research Ethics Committee of the Medical Faculty of Lund University (H4 197/2007) and was in compliance with the Declaration of Helsinki.

## Results

### Study group

In the original article by Overend et al. [10], the study group consisted of 51 patients. We tried to replicate this when making our study group, which consisted of 54 patients. Therefore, no power calculations have been performed.

The patients came from eight different health care centres in central Halland, Sweden. None of the patients had ever filled out a questionnaire concerning their forearm or elbow before. The group consisted of 54 persons: 25 women and 29 men. They all had unilateral epicondylitis/epicondylalgia. The mean age was 46 years. Nine persons were on sick leave and 45 persons were working as normal without any changes resulting from their symptoms.

### Methods

The PRTEE-S and DASH questionnaires were filled out by 54 patients. The patients were all informed in writing of the purpose of the study and that they would be given a number in order to conceal their identity. All of the patients who were asked to participate chosed to do so, and they all completed both questionnaires. There were no drop-outs.

### Test-retest reliability of the PRTEE-S

The test-retest reliability was calculated for all of the individual questions, for the separate pain and function sub-scales, and for the overall PRTEE-S score. The intraclass correlation coefficient (ICC) was high for all of the individual questions, as seen in Table 1. For the function sub-scale, the ICC was excellent (ICC's > 0.95), and for the pain subscale, the ICC was also high. The total questionnaire ICC scores (overall pain + function) was excellent (ICC's > 0.95). The test-retest correlation showed the highest reliability for question 12 (0.99) on the function sub-scale and the lowest for question 5 (0.88) on the pain sub-scale (Table 1). The highest intraclass correlation coefficient for the pain-subscale was found for question 4, "opening a jar" (0.96), and for the function sub-scale question 12, "personal care activities (i.e., dressing and washing)" (0.99). In the overall score, where the pain and function sub-scales are combined, the ICC was excellent (ICC's > 0.95). The correlation coefficients for the pain and function sub-scales and the overall score are shown in Table 2.

**Table 1:** Internal consistency.

Pain scale			Function scale	
Question	ICC		Question	ICC

1	0.94		6	0.95
2	0.94		7	0.95
3	0.93		8	0.96
4	0.96		9	0.92
5	0.88		10	0.97
			11	0.97
			12	0.99
			13	0.97
			14	0.95
			15	0.95
				
Pain			Function	
Sum scale	0.78		Sum scale	0.95
		n = 54	Overall score	0.95

Intraclass correlation coefficients (ICC) of the test-retest reliability of the PRTEE-S (Patientskattad Utvärdering av Tennisarmbåge).

**Table 2:** Reliability of the PRTEE-S (Patientskattad Utvärdering av Tennisarmbåge).

		Occasion 1	Occasion 2
Pain section	Mean Score ± SD	4.18 ± 1.81	3.77 ± 1.80
	SEM	0.25	0.25
	95% CI	0.21 – 0.78	0.23 – 0.79
	Cronbachs α	0.84	0.83
	ICC	0.58	0.60
			
Function section	Mean Score ± SD	3.90 ± 2.38	3.70 ± 2.29
	SEM	0.32	0.31
	95% CI	0.85 – 0.94	0.84 – 0.94
	Cronbachs α	0.93	0.92
	ICC	0.91	0.90
			
Overall (pain + function)	Mean Score ± SD	4.04 ± 2.00	3.74 ± 1.97
	SEM	0.27	0.27
	95% CI	0.84 – 0.94	0.84 – 0.94
	Cronbachs α	0.94	0.94
	ICC	0.90	0.90

Comparison of the PRTEE-S score for patients (n = 54) on two occasions, on the two sub-scales (pain and function score) and the total questionnaire (overall score).

### Internal reliability (internal consistency) of the PRTEE-S

The Cronbach's alpha coefficients were high for both the pain and function sub-scales (0.84 and 0.93, respectively). The coefficient for the overall PRTEE-S was excellent (0.94), as seen in Table 2.

### Construct/concurrent validity of the PRTEE-S

The pain sub-scale, the function sub-scale, and the overall score from the PRTEE-S each showed significant correlations with the DASH score (p < 0.0001) as shown in Table 3.

**Table 3:** Construct/concurrent validity of the PRTEE-S.

PRTEE-S	Pain		Function		Overall	
	r	p	r	p	r	p

DASH symptoms (questions 24–29)	0.79	<0.0001	0.83	<0.0001	0.84	<0.0001
DASH function (questions 1–21)	0.82	<0.0001	0.90	<0.0001	0.91	<0.0001
DASH overall (questions 1–30)	0.78	<0.0001	0.90	<0.0001	0.88	<0.0001
Roles & Maudsley	0.67	<0.0001	0.79	<0.0001	0.78	<0.0001

Spearman's correlation (r) was used in the calculation of the correlations between the PRTEE-S (Patientskattad Utvärdering av Tennisarmbåge), DASH (Disability for the Arm, Shoulder and Hand) and Roles & Maudsley scores.

The PRTEE-S sub-scales also showed significant correlation with the DASH score when the DASH score was divided into symptom questions (questions 24–29) in order to correlate it with the pain sub-scale (questions 1–5). The DASH score was also divided into a function sub-scale (questions 1–21) in order to correlate it with the PRTEE-S function sub-scale (questions 6–15). This had been done recently in a German cross-cultural adaptation, reliability, and validity study of the PREE (Patient-rated Elbow Evaluation) questionnaire [25], which is similar to the PRTEE. The results were all significant (p < 0.0001) [26].

The correlations between the PRTEE-S and the Roles & Maudsley score were also significant in both the pain and the function sub-scales as well as in the overall score (p < 0.0001).

## Discussion

The PRTEE-S has been cross-culturally adapted for patients in Sweden and translated into the Swedish language. The PRTEE-S was tested and considered a reliable and valid instrument for use in patients with lateral epicondylitis/epicondylalgia.

Even though the diagnosis of lateral epicondylitis/epicondylalgia is common, there is no specific instrument in Sweden that has been translated and adapted for Swedish culture that can evaluate treatment and indicate whether there are any changes in symptoms because of the treatment. The DASH is the closest questionnaire for evaluating elbow disease, but it is not as specific as the PRTEE-S. The DASH questionnaire includes the whole arm and the shoulder, whereas the PRTEE-S only evaluates the elbow. Newcomer et al. [14] suggested that the original PRFEQ is reliable, reproducible, and sensitive in the assessment of lateral epicondylitis/epicondylalgia. They correlated the PRFEQ with the VAS, DASH, pain-free grip and SF 36, and found that the PRFEQ was at least as sensitive to changes as other commonly-used outcome tools, but its advantage lies in the focus on the elbow and therefore should be used as a standard outcome measure in all lateral epicondylitis/epicondylalgia research. Another questionnaire, called the LES (Liverpool Elbow Score), is used in Liverpool [27], but this is not a totally patient-rated questionnaire. It also contains some clinical data, making it harder to use if the purpose in the research or clinical setting (or possibly even by mail) is to get a quick response from the patient. The LES has been developed in a tertiary care setting and has not been tested in a primary care setting.

Another advantage of the PRTEE-S is the short time that it takes to fill it out: can be completed in only a few minutes. If the PRTEE-S is used in a scientific report, it can easily be sent by mail to the responder. It is also very easy for the therapist to evaluate. As far as we know, there is no other elbow-specific questionnaire for Swedish or even Scandinavian language-speaking people.

The PRTEE-S measures the pain and function over the previous week, which is another advantage. This disease often changes because of a person's activities and occupation. When the therapist sees the results of the questionnaire, he/she can easily decide if there is a need for ergonomic support in the patient's work, home, or recreational activities.

Although it measures several different movements of the elbow, the PRTEE-S can be hard to fill out if the dominant elbow is not the one that is measured. For example, consider a patient who suffers from a lateral epicondylitis in her right arm, but is left-hand dominant. For her, it may be hard to fill out the questionnaire, as she usually does not use her right arm to "opening a jar". Another disadvantage of the PRTEE-S is that if the patient does not perform several of the tasks, the results of the questionnaire can be misleading.

The process of translating and back-translating the English PRTEE [16] was carried out according to the guidelines of Beaton's [18] five steps. This is an easy and well-described way to perform a cross-cultural adaptation. If the steps are carefully followed, the cross-cultural adaptation is consistent in the content and face validity between the source and target versions of the questionnaire. Therefore, it should follow that if the original version was reliable and valid, the translation should be as well. Rompe [16] validated the updated version [15] of the PRTEE and found it to be valid and as sensitive to changes as the previous PRFEQ questionnaire [10]. However, Dr Overend, who was the first to publish the reliability of the scale, used a mean item scoring method. The method recommended by the developer is the 100-point method where the maximal pain section score would be 50 points. The function section is 100 points but is divided by 2, so this section is also worth 50 points; each section then contributes equally to the overall score of 100 points. The 100 point-method is easier and allows one to compare the number using a metric that is compatible with other commonly used scales.

The expert committee who performed the cross-cultural adaptation and translated the English version into Swedish did not make any major changes, and all members agreed that the final version was easy to understand and correlated well with the original version.

The usual duration between a patient's first and second visit for both the physiotherapist and the occupational therapist is a week. This seemed too long, as the symptoms could change over time [28]. However, a limitation of a short interval between the two occasions in which the patients filled out the forms could be that they remembered their original answers the second time. The patients who answered the questionnaires were almost equally distributed between genders, with 25 women and 29 men. There were no differences according to gender.

The Swedish PRTEE-S version showed good reliability overall on the individual test-retest questions, with high values on the ICC, as the coefficients were between 0.88 – 0.99. Therefore, for the test-retest, all of the individual questions were considered to be excellent. The mean of the pain scores of the PRTEE-S were 4.18 ± 1.81, which is almost identical to the mean of 4.1 ± 1.8 reported in the original article [10]. In this article, the ICC for the pain section was 0.58, which is a bit lower than in the original (0.94), but is still fair to good.

When comparing the PRTEE-S function scores to the function scores in the original article, the ICC was higher for the PRTEE-S (0.91). The original article had an ICC of 0.83. The entire (overall) score for the PRTEE-S was 0.90, which is almost identical to the original (0.89). The Cronbach's alpha for the function sub-score (0.93) and the overall score (0.94) was very high. This indicates that the internal consistency of the questionnaire was very high. When a questionnaire is used in a clinical setting, an alpha coefficient of at least 0.9 is recommended [29]. The Cronbach's alpha for the pain subscale (0.84) is considered to be very close to the recommended score.

To assess the criterion/construct validity of the PRTEE-S, correlations were made among the PRTEE-S, the DASH questionnaire and the Roles & Maudsley score. When comparing the PRTEE-S with the DASH questionnaire, we decided to use a German cross-cultural adaptation of a similar questionnaire (PREE) that had recently been completed [26]. The DASH questionnaire measures the whole arm and shoulder, and therefore has slightly different questions. For that reason, we decided to divide the questionnaire into two sections; the DASH symptom section consisted of questions 24–29, and the function section consisted of questions 1–21. These two sections, as well as the entire DASH questionnaire, were correlated with the PRTEE-S. The symptom score of the DASH questionnaire, which also included three pain questions, showed a high correlation (r = 0.79), while for the German PREE-G questionnaire, this score was r = 0.61. The combined scores from the PRTEE-S showed an even higher correlation with the DASH questionnaire (r = 0.88) and a high correlation for the PREE-G (r = 0.73). There were also high correlation scores in the function sub-scale (r = 0.90). For the PREE-G, this correlation was 0.83. The results were all significant (p < 0.0001). The highest correlation was found between DASH function and the overall score (r = 0.91). When we used the Roles & Maudsley score to correlate the PRTEE-S, we found that it correlated moderately in the pain sub-scale (r = 0.67), more in the overall scale (r = 0.78), and most in the function sub-scale (0.79).

The use of the PRTEE-S is a way to quickly estimate patients' self-reported problems. It is also a very inexpensive way to evaluate changes in a patient's rehabilitation progress, and can be used in research papers. It has now been translated from English into two languages [10,17,26], and perhaps there will be more translations in the future. This would make it easier to compare the results from different research articles.

## Conclusion

The PRTEE-S is a reliable and valid instrument to evaluate subjective outcomes in Swedish-speaking patients with lateral epicondylitis/epicondylalgia and can be used in both research and clinical settings.

## Competing interests

The authors declare that they have no competing interests.

## Authors' contributions

PN participated in the design of the study, data collection and analysis, and writing of this manuscript. AB participated in the analysis. BM and JM participated in the analysis and writing of this manuscript. All authors read and approved the final manuscript.

## Pre-publication history

The pre-publication history for this paper can be accessed here:



## Supplementary Material

Additional file 1 PRTEE-S questionnaire. The data provided is the Swedish version of the PRTEE questionnaire (Patientskattad Utvardering for Tennisambage). Click here for file
