# Supplementary material for: Cross-cultural adaptation and determination of the reliability and validity of PRTEE-S (Patientskattad Utvärdering av Tennisarmbåge), a questionnaire for patients with lateral epicondylalgia, in a Swedish population
Source: BMC Musculoskelet Disord. 2008 Jun 5;9:79. doi: 10.1186/1471-2474-9-79 (PMC2435532; doi:10.1186/1471-2474-9-79)
Supplement: Additional file 1 — PRTEE-S questionnaire. The data provided is the Swedish version of the PRTEE questionnaire (Patientskattad Utvardering for Tennisambage). [file 1471-2474-9-79-S1.doc]

**Additional file 1**

**PRTEE-S (PATIENTSKATTAD UTVÄRDERING AV TENNISARMBÅGE)**

Frågorna nedan hjälper oss att förstå hur mycket problem du har haft med din arm under den senaste veckan. Du skall beskriva dina **genomsnittliga** symptom gällande din arm **under den** **senaste veckan** på en skala från 0-10. Var snäll och svara på alla frågorna. Om du inte gjort en aktivitet pga smärta eller att du inte klarat av aktiviteten så ska du markera ”10”. Om du är osäker så gör en skattning så gott du kan. Låt bara bli att svara på en fråga om du aldrig utför aktiviteten. Markera detta genom att dra en linje helt igenom frågan.

1. SMÄRTA i din skadade arm

Var god skatta den genomsnittliga smärtan i din arm **den senaste veckan** genom att ringa in den siffran som bäst beskriver din smärta på en skala från 0-10. En **nolla** (0) betyder att du **inte haft någon smärta** och en **tia** (10) betyder **värsta möjliga smärta**.

UPPSKATTA DIN **SMÄRTA**

Ingen smärta Värsta tänkbara smärta

Vid vila 0 1 2 3 4 5 6 7 8 9 10

Vid upprepade armrörelser 0 1 2 3 4 5 6 7 8 9 10

Bära en påse med matvaror eller 0 1 2 3 4 5 6 7 8 9 10

portfölj i handtaget

När smärtan kändes som minst 0 1 2 3 4 5 6 7 8 9 10

När smärtan kändes som mest 0 1 2 3 4 5 6 7 8 9 10

Vg vänd blad

2. FUNKTIONSNEDSÄTTNING

A. SPECIFIKA AKTIVITETER

Uppskatta **hur stora svårigheter** du upplevde vid utförandet av de olika aktiviteterna nedan, under den senaste veckan, genom att ringa in den siffran som bäst beskriver dina svårigheter på en skala från 0-10. En **nolla** (0) betyder att **du inte hade några svårigheter** och en **tia** (10) betyder att **du inte kunnat utföra aktiviteten alls**.

UPPSKATTA DIN **FUNKTION**

Klarar utan svårighet Kan ej utföra pga smärta

Vrida om dörrhandtag eller nyckel 0 1 2 3 4 5 6 7 8 9 10

Bära en påse matvaror

eller portfölj i handtaget 0 1 2 3 4 5 6 7 8 9 10

Lyfta en fylld kopp kaffe

eller ett glas mjölk till din mun 0 1 2 3 4 5 6 7 8 9 10

Öppna en burk 0 1 2 3 4 5 6 7 8 9 10

Dra på dej byxorna 0 1 2 3 4 5 6 7 8 9 10

Vrida ur tvättade kläder

eller en blöt handduk 0 1 2 3 4 5 6 7 8 9 10

B. VANLIGA AKTIVITETER

Uppskatta **hur stora svårigheter** du upplevt i dina vardagliga sysselsättningar i frågorna nedan under den senaste veckan, genom att ringa in den siffran som bäst beskriver dina svårigheter på en skala från 0-10. Med vardagliga sysselsättningar menar vi sådana aktiviteter du utfört **innan** du fick problem med din arm. En **nolla** ( 0 ) betyder att **du inte har några svårigheter** och en **tia** ( 10 ) betyder att **du varit oförmögen att utföra aktiviteten**.

UPPSKATTA DIN **FUNKTION**

Klarar utan svårighet Kan ej utföra pga smärta

Personlig vård (klä sig, tvätta sig) 0 1 2 3 4 5 6 7 8 9 10

Hushållsarbete (städning) 0 1 2 3 4 5 6 7 8 9 10

Arbete (ditt jobb eller vardagsarbete) 0 1 2 3 4 5 6 7 8 9 10

Fritids- eller idrottsaktiviteter 0 1 2 3 4 5 6 7 8 9 10

Kommentarer: ……………………………………………………………………………….

**Instruktioner för PRTEE-S (Patientskattad Utvärdering för Tennisarmbåge)**

Minimera antalet obesvarade frågor genom att kontrollera när patienten är klar med ifyllandet. Säkerställ att patienten som lämnat en rad blank för att han/hon ej kunnat utföra funktionen, förstått att detta skulle ha fyllts i som ”10”. Rätta om nödvändigt.

Om patienten är osäker om vilken siffra som skall väljas för att han/hon utfört någon aktivitet under senaste veckan, skall siffran väljas som närmast motsvarar hans/hennes medeltal av svårighet att utföra funktionen. Detta blir mer rätt än att lämna raden blank. Däremot om han/hon *aldrig* utför en funktion/aktivitet är det bättre att lämna raden blank. Om raden från en fråga lämnas blank tas ett medeltal av de övriga frågorna i kolumnen för t.ex smärta för denna rad. Både summan av kolumnerna för sig och den totala summan för hela enkäten har hög reliabilitet vilket innebär att båda sätten är användbara för utvärdering.

**Smärta**

Summera upp till fem frågor Bäst poäng = 0 Sämst poäng = 50

**Specifika aktiviteter**

Summera upp till sex frågor Bäst poäng = 0 Sämst poäng = 60

**Vanliga aktiviteter**

Summera upp till fyra frågor Bäst poäng = 0 Sämst poäng = 40

**Funktion kolumn**

(Specifika aktiviteter + Vanliga aktiviteter)/2 Bäst poäng = 0 Sämst poäng = 50

**Total poäng** = Smärta kolumn + Funktion kolumn

Bäst poäng = 0 Sämst poäng = 100 (smärta och funktionsnedsättning bidrager lika till totalsumman).
